# Effect of modified esophagectomy perioperative technique resection for patients with locally advanced esophageal cancer (tumor length > 8 cm): initial experience in 45 cases

**DOI:** 10.1186/s13019-022-01942-3

**Published:** 2022-09-02

**Authors:** Yunfei Wu, XuWu, Junhua Zhang, Xiang Li, Nanbo Liu, Jun Li, Xuyuan Chen, Lichun Wei

**Affiliations:** 1 Department of Thoracic Surgery, Huiqiao Medical Center, Nanfang Hospital, Southern Medical University, No. 1838, North Guangzhou Road, Guangzhou, Guangdong China; 2 Department of Thoracic Surgery, The Affiliated Hospital of Southwest Medical University, Luzhou, China

**Keywords:** Esophageal cancer, Esophagogastric fistula, Length, Locally advanced esophageal cancer esophagotracheal fistula, Modified (Wu’s) esophagectomy

## Abstract

**Background:**

Patients with locally advanced esophageal cancer with a lesion length greater than 8 cm (LCWEC) are prone to high mortality in a short time due to esophagotracheal fistula (ETF) and esophagoaortic fistula (EAF). We tried to explore a safe salvage surgical method during the perioperative period to maximize the resection of the tumor on the premise of safety and reconstruction of the alimentary tract to avoid early death due to ETF and EAF.

**Methods:**

From December 2007 to November 2018, forty-five LCWEC patients were treated using the modified Wu’s esophagectomy. Patient features, surgical techniques, postoperative complications, and pathology outcomes were analyzed.

**Results:**

The average length of the tumors was 12.5 cm (range 8.1–22.5 cm), and the average transverse tumor diameter was 5.8 cm (range 4.5–7.8 cm). No complications like anastomotic leakage, anastomotic stenosis, chylothorax, delayed gastric emptying, vocal cord paralysis, dumping syndrome, and reflux were detected. The 30-day and in-hospital mortality rates were 0%. Complete (R0) resection was achieved in 38 (84.4%) cases. The resection margin rate of positive anastomosis was 0%. Until the death of the patients, no feeding failure due to gastrointestinal obstruction and early death due to ETF or EAF occurrence. During follow-up, the median time to death was 17.2 months for patients treated with surgery alone and 32 months for patients treated with postoperative multimodal treatment.

**Conclusion:**

The modified Wu’s esophagectomy is a safe and feasible salvage surgical method for LCWEC resection.

## Background

In China, locally advanced esophageal cancers with a length greater than 8 cm (LCWEC) account for 15.7–25.5% of the patients receiving treatment for esophageal cancer [1–3]. These cases are commonly staged T3 or T4, without tumor metastasis in important systemic organs and with or without tumor metastasis in supraclavicular and abdominal lymph nodes. Moreover, the patients are without hematemesis symptoms.

**Fig. 1:**
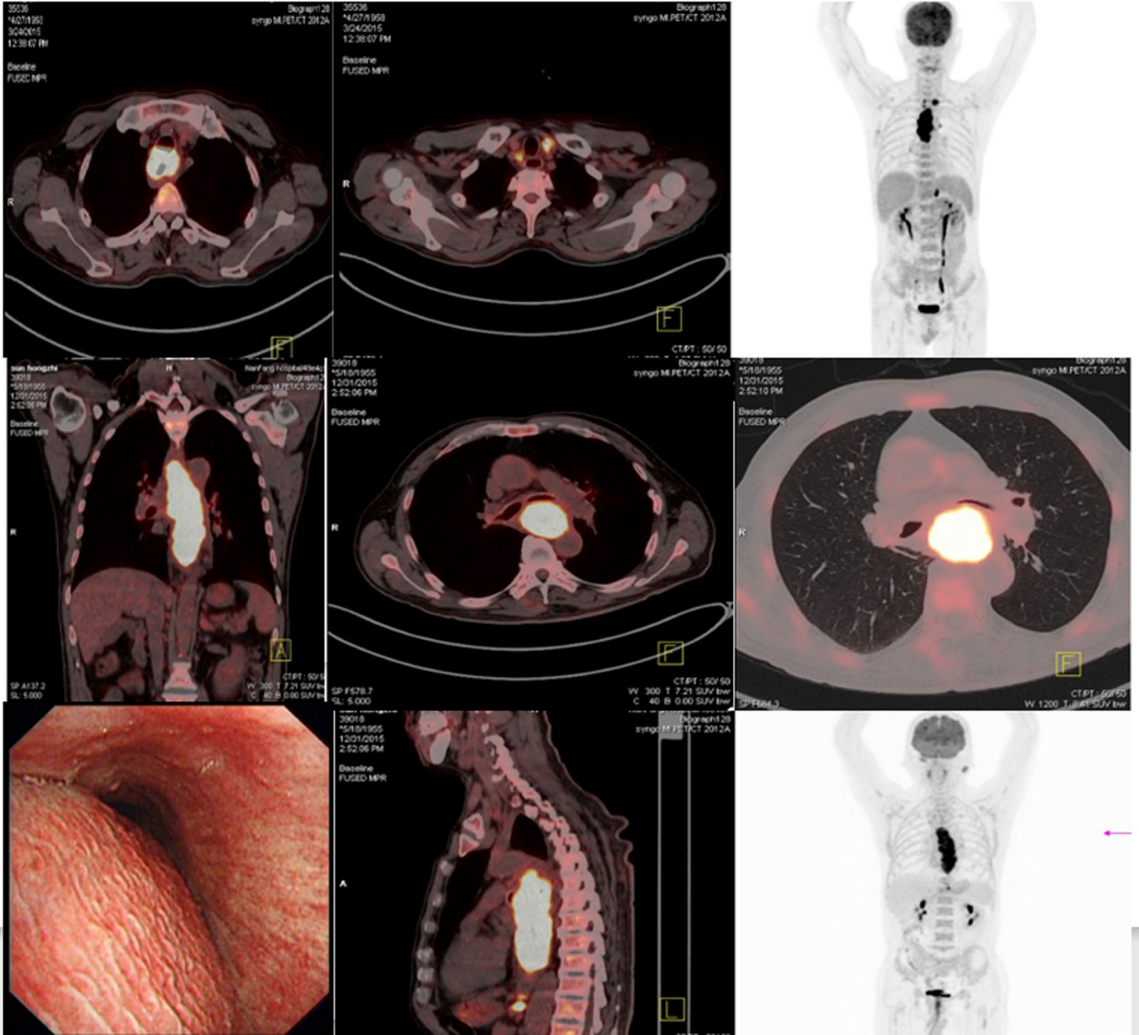
Imaging manifestations of LCWEC. PET showing the initial invasion of the esophageal cancer tumor of the tracheal membrane and the aorta, with almost complete obstruction of the esophagus and compression of the trachea, without tumor metastasis in important systemic organs, with or without tumor metastasis of supraclavicular and abdominal lymph nodes

The tumors (Fig. 1) start their invasion of the trachea and the aorta. The tumors do not usually completely invade the trachea and the aorta and cannot thus be removed. Additionally, such cases have high mortality within a short time due to dangerous and serious complications such as complete esophageal obstruction, tracheal obstruction, aspiration pneumonia, esophagotracheal fistula (ETF), and esophagoaortic fistula (EAF) [4–8]. Although patients can receive palliative treatments such as radiotherapy, chemotherapy, aortic stent, esophageal stent, and gastrostomy, they cannot eliminate the dangerous complications caused by the tumor and improve the quality of life [4, 9–14].

Surgical resection is still the cornerstone for managing locally advanced esophageal cancer, but the safe resection and removal of the tumor with few complications and limited possibility of the residual tumor remains controversial. Therefore, in this study, we aimed to explore a safe salvage surgical method during the perioperative period to maximize the resection of the tumor on the premise of safety and reconstruction of the alimentary tract to avoid early death due to ETF and EAF. The purpose of this method was also the improvement of the patients’ quality of life, the reduction of mortality, the prolongation of survival time, the increase of the long-term survival rates, and saving valuable time for subsequent comprehensive treatment. We performed a modified McKeown esophagectomy, and here, we report our findings thus far (referred to as Wu’s esophagectomy).

## Materials and methods

### Patient selection

**Fig. 2:**
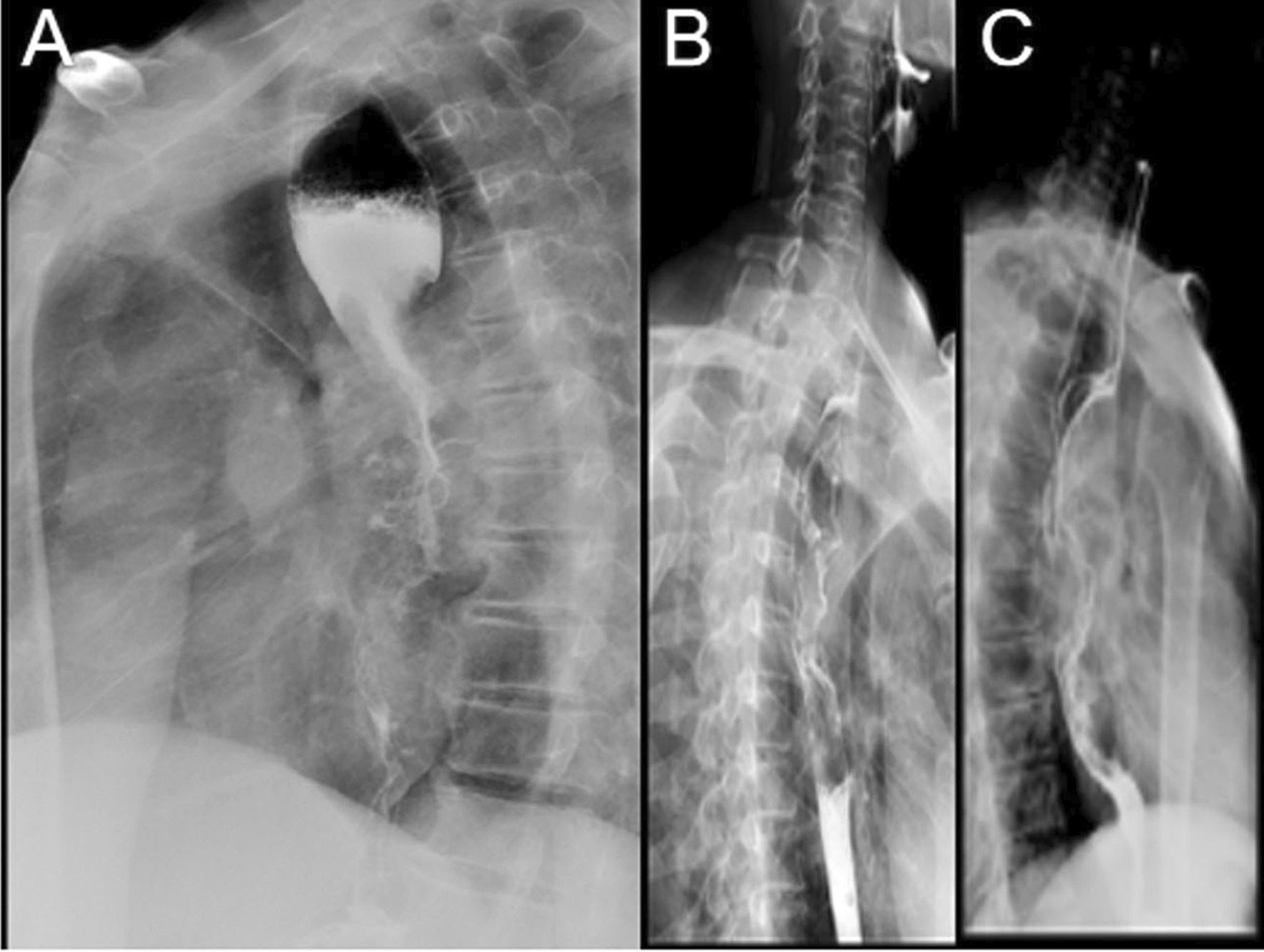
Preoperative upper gastrointestinal radiography manifestations of LCWEC. Preoperative upper gastrointestinal radiography displayed the filling defects caused by esophageal cancer in images **A**, **B** and **C**, which were 22.5 cm, 12.6 cm, and 18.3 cm, respectively, with almost complete obstruction of the esophagus

This retrospective study analyzed the patients who underwent modified McKeown resection of esophageal cancer at the Southern Hospital of the Southern Medical University (Guangzhou, Guangdong, China) from December 2007 to November 2018. Preoperative examinations were performed, including gastroscopy, upper gastrointestinal radiography (UGR), fiberoptic bronchoscopy, contrast-enhanced computed tomography (CT) of the neck, chest, and abdomen, and positron-emission computed tomography (PET-CT) examination. For inclusion, the patients had to meet the following conditions: (1) esophageal lesions evaluated by UGR with a length greater than 8 cm (Fig. 2); (2) the pathological diagnosis of the esophageal lesions was squamous cell carcinoma; (3) PET-CT showed no distant metastasis in the brain, lungs, liver, kidneys, and other important organs; (4) CT revealed no obvious invasion of the esophageal tumor into the adjacent structures of the trachea and aorta; (5) three-dimensional CT reconstruction of the aorta showed that the vessels of the aortic branches were not enlarged and grew into the esophageal tumor; (6) fiberoptic bronchoscopy showed no invasion of the posterior tracheal wall and no tumor cells were detected on the tracheal brush; (7) the anesthesiologists assessed lung function to maintain single-lung ventilation; (8) no serious comorbidities such as cirrhosis or renal failure; (9) Eastern Cooperative Oncology Group Performance Status (ECOG PS) score ≤ 2; (10)and without hematemesis or hemoptysis.

### Surgical procedures

All patients underwent four stages of esophagectomy and alimentary tract reconstruction. The right lung was collapsed during thoracoscopic surgery. All patients were given analgesic epidural anesthesia.

In the first phase, the patient was placed in the left lateral decubitus position. A 10-mm port was made in the 7th intercostal region of the right midaxillary line for observation. An 8-mm port was placed at the fifth intercostal space on the lateral side of the right chest as the operating port to explore and clarify the scope of the tumor and the feasibility of surgical resection, and the thoracic cavity was closed.

In the second phase, the surgeon stood on the patient’s right side, and the patient was in the supine position. The laparoscope was inserted through a subumbilical incision to explore the abdominal cavity. Next, a 10-mm trocar at the left midclavicular line at the horizontal of the umbilicus was used for laparoscopic instruments. A 5-mm trocar was placed at the left midclavicular line below the costal edge, which could be used for the surgical assistant. The Lap Disc (Ethicon Endo-Surgery, Cincinnati, OH, USA) was arranged through a midline incision approximately 7 cm below the xiphoid. With the assistance of the left hand, an Ultracision Harmonic Scalpel (Ethicon Endo-Surgery, Cincinnati, OH) was used to mobilize the stomach, and a 5-cm segment of the esophagus was dissociated through the hiatus.

The esophagus was cut off with an Echelon Flex 60 linear stapler (Ethicon endo-surgery, Cincinnati, OH, USA). The esophagus’s proximal end was sutured with a double thread, and the esophageal hiatus was sutured with two stitches. The diaphragmatic, the para-cardia, the left gastric, the proximal common hepatic artery, the proximal splenic artery, and the celiac trunk lymph nodes were removed. The stomach was pulled out of the abdominal cavity. A sufficient amount of tissue had to be removed around the pylorus to prevent pylorus angulation. Most importantly, partial pyloromyotomy was performed instead of pylorotomy. The muscular layer of the strongest pyloric ring sphincter was longitudinally cut at an approximative length of 1.5 cm. Next, the muscular layer was evenly and transversely sutured with three intermittent sutures. Next, a gastric tube with an approximate width of 4–5 cm was made. It was the first extension of the gastric tube, which was usually sufficient to complete a cervical anastomosis. If the gastric tube was not sufficiently long, the gastric tube was lengthened. Several triangles were cut at a suitable angle and distance from the lesser curve of the stomach with an Echelon Flex 60 Flex linear stapler (Ethicon endo-surgery), according to the length that needed to be extended. The final step in this phase was the arrangement of a feeding jejunostomy tube.

After the abdominal operation, the left recumbent position was taken for the chest operation in the third stage. A 10-mm operation port was arranged at the second and fifth intercostal spaces, just in the midaxillary line. After the mediastinal pleura above the thoracic esophagus was incised, the azygos vein was cut off with an Echelon Flex 60 linear stapler (Ethicon Endo-Surgery). The lower thoracic esophagus was completely dissected manually to avoid injury to the azygos vein, the inferior vena cava, and the descending aorta. The suture of the lower esophagus was touched and grasped. The esophagus of the upper thoracic cavity was mobilized with the fingers, and the esophagus was transected close to the upper thoracic cavity with an Echelon Flex 60 Flex linear stapler (Ethicon endo-surgery). The esophagus was carefully dissected along the edge of the tumor. The ligamentous connective tissue of the posterior wall of the esophagus was ligated during the mobilization to avoid chylothorax. Special attention was paid to the close anatomical relationship between the esophageal tumors and the tracheal membranes or the aorta and possible iatrogenic injuries during the operation. Under manual palpation, the boundary between the periphery of the esophageal tumors and the tracheal membrane or the aorta was slowly bluntly dissected with the index finger. For the management of the close relationship between esophageal tumor and the trachea, the aorta, and the lymph nodes of the recurrent laryngeal nerve, similar surgical strategies were adopted. If the esophageal tumor closely adhered to the tracheal membrane or the aorta and could not be completely removed, the periphery of the esophageal tumor was carefully dissected. Finally, the involved aorta and tracheal membrane were dealt with. Palliative resection was performed with a sharp resection between the tracheal membrane, the aorta, and the esophageal tumor by the esophageal side. Rough and blunt separation was not performed, and the tracheal membrane and aorta were not damaged.

After the tumor was resected, the enlarged mediastinal lymph nodes were removed as far as possible, and the recurrent laryngeal nerve lymph nodes were eliminated without using energy instruments (harmonic scalpel—Ultracision, Electric hook, or electric knife). If the recurrent laryngeal nerve was closely adherent to the lymph nodes and could not be completely resected, sharp palliative resection was performed between the lymph nodes and the recurrent laryngeal nerve on the side of the lymph nodes. An argon beam coagulator (Beamer plus, ConMed Corporation, Utica, NY, USA) was used to deal with extensive blood oozing, and an electric knife was used to treat blood oozing from the tiny blood vessel. The Ultracision harmonic scalpel (Ethicon Endo-Surgery) was used to precisely deal with the branch of blood vessels entering the tumor. Hot water (43 °C) gauze was used to press the site to reduce bleeding and promote clotting.

The following pretreatment measures were taken against possible residual lesions after the tumor resection: soaking of the chest cavity with distilled water, smearing the lesion with 2% iodine solution, flaming burns lesions directly with an argon beam coagulator (Beamer plus, ConMed Corporation, USA), and soaking of the lesions in elemene emulsion (400 mg dose) (Jingang Pharmaceutical Co, Dalian, China). The thoracic cavity was closed.

The fourth stage of neck surgery was performed in the supine position. After the xiphoid process was resected and a posterior sternal tunnel was made by blunt dissection, the gastric tube was pulled to the left side of the neck. A 7-cm incision was made in the anterior marge of sternocleidomastoid. The left sternothyroid, sternohyoid, and omohyoid muscles near the side of the sternum were mobilized with the index finger and divided with electrocautery. Then, the cervical esophagus was separated and exposed with the index finger to avoid the left recurrent laryngeal nerve injury with sharp instruments. In order to find the cervical esophagus, the index finger was used to follow the tracheoesophageal groove and enter the thoracic inlet. The upper inner thoracic esophagus was grasped and was finally pulled out. A curved intraluminal stapler CDH25A (USA) was used to perform an end-side anastomosis between the cervical esophagus and the gastric tube by a modified technique. One important modification of the anastomotic pattern was that the anvil of the circular stapler on the esophagus’s side and the esophageal lumen’s cross-sectional diameter remained fixed at 30°. The divided neck muscle was sutured. Another important modification for preventing cervical anastomotic leakage included placing drainage tubes on both sides of the neck anastomotic site and three intermittent loose sutures for the cervical incision. Routine tracheal intubation was done in the intensive care unit (ICU), and a ventilator was routinely used for 3 days after the surgery. Oral feeding was started 9 days postoperatively.

### Data collection

The basic demographic and comorbidity data were collected. The following perioperative data were collected: total operative time, transfusion, blood loss, duration of the intensive care unit stay, length of the hospital stay, in-hospital mortality within 30 days, and complications. The tumor variables included tumor stage, tumor length, the maximum transverse diameter of the tumor, histology, R0 resection, circumferential margins (CRMs), the cutting edge of positive anastomosis, and the number of harvested lymph nodes. Positive circumferential margin (CRM) was defined as the presence of tumor cells within 1 mm of the transverse margin of esophageal cancer lesions. The clinicopathological stages were assessed according to the 8th UICC-AJCC esophageal TNM staging system [15]. The complications were defined based on the Clavien-Dindo (CD) and the Esophagectomy Complications Consensus Group (ECCG) [16–18]. The postoperative anastomotic leakage and the width of the anastomotic orifice were determined by radiology (iodohydrin contrast UGR under DSA) 8 days after surgery. Anastomotic stenosis was defined as clinically significant dysphagia caused by anastomotic stenosis and the width of the anastomotic orifice on the UGR orifice [19]. The gastric emptying test was conducted to determine delayed gastric emptying (DGE) using 200 mL of methylene blue diluted water solution, drunk every 20 min within 1 h, equivalent to 600 mL per hour. A gastric tube was placed to measure the gastric retention fluid next hour. Reflux symptoms were defined as heartburn (retrosternal heartburn) and regurgitation (material and acid taste in the mouth moving up from the stomach) [20]. After surgery, all patients were instructed to continue and complete multi-mode therapy (including radiotherapy, chemotherapy, and/or immunotherapy) and were followed up regularly. In addition, some patients were given immunotherapy and chemotherapy therapy in clinical trials.

### Statistical methods

SPSS 24.0 (SPSS Inc., Chicago, IL, USA) was used for the statistical analysis.

## Results

### Demographic characteristics

From December 2007 to November 2018, 45 patients at the Southern Hospital of the Southern Medical University (Guangzhou, Guangdong, China) underwent modified (Wu’s) esophagectomy for LCWEC. The demographic and clinical characteristics are presented in Table 1. There were 13 females and 32 males, with a median age of 59.6 years (range 42–78 years). One patient had received radiotherapy and chemotherapy before surgery. There were 10 cases (22.2%) with tumors located in the upper segment, 30 (66.7%) with tumors in the middle segment, and five (11.1%) with tumors in the lower segment. All patients had a preoperative pathological diagnosis of esophageal squamous cell carcinoma.

**Table 1:** Patient characteristics

	All number (n = 45) (%)
Age (years), mean (range)	59.6 (42–78)
Sex (M/F)	
Male	32 (71.1%)
Female	13 (28.9%)
Body mass index (BMI), mean ± SD	19.15 ± 2.38
Blood routine test	
Hemoglobin (g/L), mean ± SD	118.8 ± 26.64
Albumin (g/L), mean ± SD	33.1 ± 1.9
History of smoking	35 (77.7%)
History of alcohol	28(62.2%)
Prior gastric or esophageal surgery	0 (3.2%)
Previous chest surgery	1 (1.6%)
Comorbidities	
Coronary artery disease	3 (6.0%)
Diabetes mellitus	11 (24.4%)
COPD/emphysema	4 (8.8%)
Location of lesion	
Upper thoracic esophagus	10 (22.2%)
Middle thoracic esophagus	30 (66.7%)
Lower thoracic esophagus	5 (11.1%)

### Oncologic outcomes

The tumor parameters are shown in Table 2. UGR revealed that the length of esophageal lesions was greater than 8 cm. The mean length of the esophageal tumors was 12.5 cm (range 8.1–22.5 cm), and the mean maximum transverse diameter of the tumor was 5.8 cm (range 4.8–7.8 cm). Preoperative examination (CT and PET-CT scan) established that the tumors had not invaded their adjacent structures and could be removed. R0 resection was achieved in eight (17.7%) patients, and the circumferential margins of anastomosis were all negative. The pathological stages ranged from T3N1M0 to T4bN3M1. G1 and G2 were the only two types of pathological differentiation, accounting for 15 (33.3%) and 30 cases (66.7%), respectively. III B, IV A, and IV B were the only three types of postoperative pathological staging, accounting for 19 (42.2%), five (11.1%), and 21 cases (46.7%), respectively.

**Table 2:** Pathologic outcomes

	All (n = 45) (%)
Tumor length (cm), mean ± SD	12.5 ± 5.0
Maximum transverse	
Diameter of tumor (cm), mean ± SD	5.8 (range 4.5–7.8)
Depth of tumor invasion	
pTis	0
pT1	0
pT2	0
pT3	5 (11.1%)
pT4a	37 (82.2%)
pT4b	3 (6.7%)
Lymph node metastasis	
pN0	0
pN1	11 (24.4%)
pN2	16 (35.6%)
pN3	18 (40.0%)
M1(supraclavicular lymph nodes)	11 (24.4%)
Pathologic stage	
0	0
1	0
2A	0
2B	0
IIIB	19 (42.2%)
IVA	5 (11.1%)
IVB	21 (46.7%)
Differentiation	
G1	15 (33.3%)
G2	30 (66.7%)
G3	0
Gx	0
R0 resection	8 (17.7%)
Positive CRM	37 (82.2%)
Cutting edge of positive anastomosis	0

### Surgical results

**Fig. 3:**
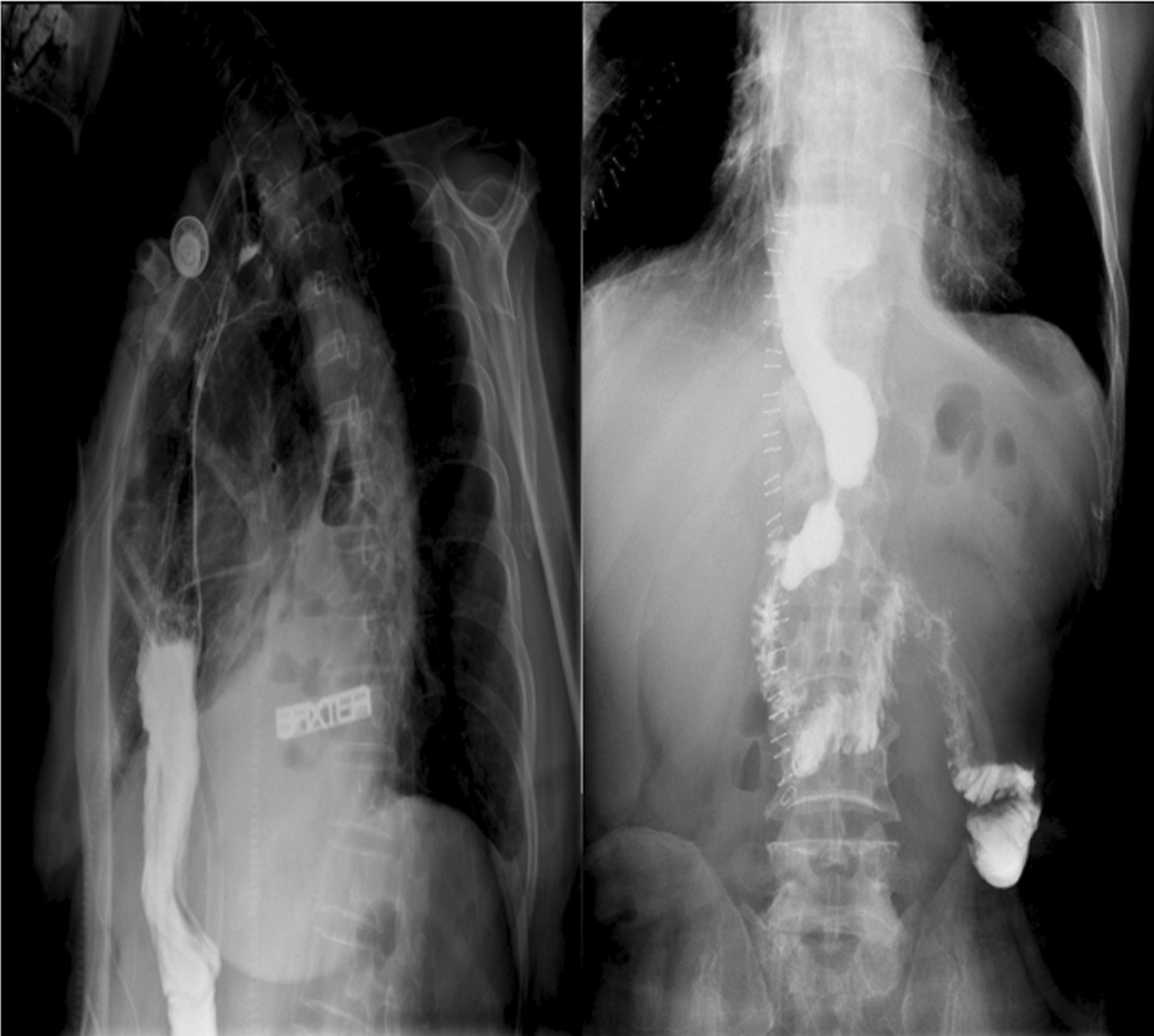
Postoperative upper gastrointestinal radiography manifestations of LCWEC. Postoperative upper gastrointestinal radiography illustrating that the gastric emptying was unobstructed

The surgical data and postoperative complications are shown in Table 3. No complications occurred, including anastomotic leakage, anastomotic stenosis, chylothorax, DGE, vocal cord paralysis, dumping syndrome, and reflux (Fig. 3). The 30-day and in-hospital mortality rates were 0%. The width of the anastomotic orifice was 21.7 ± 2.2 mm. The median blood loss was 375 mL. The median duration of stay in the ICU was 3.2 days (range 3–5 days). The length of hospitalization (LOH) was 17.5 ± 5.3 days.

**Table 3:** Intraoperative characteristics and surgical

Variables	
Operation time (min)	583 (range 286–1075)
blood loss (ml), mean ± SD	375 ± 104
Preoperative blood transfusion, n (%)	6 (13)
Intraoperative blood transfusion, n (%)	33(74)
Postoperative blood transfusion, n (%)	6 (13)
Number of harvested lymph	28.2 (8.6)
Nodes, Mean ± SD	
Thoracic	19.1 ± 6.4
Abdominal	6.5 ± 3.3
Supraclavicular	2.6 ± 1.2
Width of the anastomotic orifice (mm)	21.7 ± 2.2
Overall complications, n (%)	4 (8)
Surgical complications, n (%)	
Anastomotic leakage^a^	0
Chylothorax^b^	0
Vocal Cord Injury/Palsy^c^	0
Delayed gastric emptying α	0
Anastomotic stenosis β	0
Regurgitation^d^	0
Wound infection^e^	0
Necrosis of gastric tubeθ	0
Non-surgical complications, n (%)	
Pneumonia^f^	3 (6.0)
Atrial fibrillation^g^	1 (2.0)
Reoperation within 30 days	0
In-hospital/Mortality within 30 days	0
Postoperative length of stay (days), mean (SD)	17.5 (5.3)
Reintubation	0
(Death) DVT/pulmonary embolism	0

^a^ECCG, Type I–III; ^b^ECCG, Type I–IIII; ^c^Type I–III; αCD, Grade I–II; βCD, Grade; ^d^CD, Grade I; θ CD, Grade I; ^e^CD, Grade I; ^f^CD, Grade II–III; ^g^CD, Grade III

### Follow-up

**Fig. 4:**
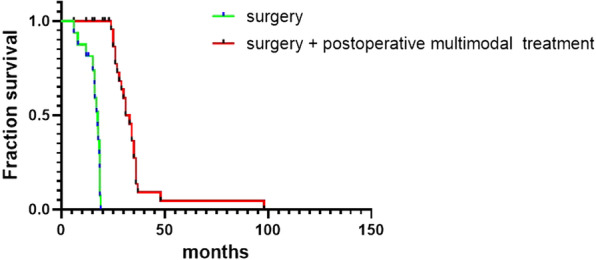
Kaplan–Meier survival estimates of multimodal treatment versus surgery for LCWEC patients

Sixteen patients did not receive any treatment after surgery (single surgery group). Twenty-nine patients completed or intermittently completed multimodal tumor treatment, including radiotherapy, chemotherapy, and immunotherapy (Fig. 4). The 6-month survival rate was 100%, the 1-year survival rate was 93%, the 2-year survival rate was 11%, the 3-year survival rate was 6%, and the 5-year survival rate was 4%. One patient was still alive in the single operation group and operation + multimodal treatment group. The patients who were unable to eat due to swallowing obstruction before surgery can eat smoothly through the mouth after surgery. Until the death of the patients, no feeding failure due to gastrointestinal obstruction and rapid death due to ETF or EAF occurrence after the surgery. All patients had distant metastasis, multiple organ failure, or local recurrence. Patients treated with multiple modalities had a significant survival benefit compared to those treated with surgery alone (Table 4 and Fig. 4). The median time to death was 17.2 months (range 0–19 months) for patients treated with surgery alone and 32 months (range 0–98 months) for patients treated with multiple modalities. The longest survival after multimodal treatment was 98 months.

**Table 4:** Pathological staging and follow-up of patients treated with either postoperative multimodal treatment or surgery alone

	Multimodal (n = 29)	Surgery (n = 16)	*P*
R0 Resection	5 (17.2)	3(18.7)	> 0.05
Positive CRM	24 (82.7)	13(81.2)	> 0.05
Pstage IIIB–IVB	29 (100.0)	16 (100.0)	> 0.05
Survival, median (range)	32 (0–98)	19.15 ± 2.38	< 0.001

## Discussion

Because of the length of LCWEC and the severity of tumor invasion, it is controversial whether LCWEC can be safely resected with few complications. We argue that the T4b staging of the eighth edition AJCC/UICC esophageal cancer TNM stage still should be further subdivided [16]. According to clinical practice, we suggest that T4b staging should be subdivided into two degrees: complete tumor invasion and the tumor is beginning to invade the trachea and aorta. For the latter, it is possible to resect the tumor, especially using the modified (Wu’s) esophagectomy. On the other hand, it is generally believed that LCWEC with or without tumor metastasis of supraclavicular lymph node and abdominal lymph node belongs to advanced esophageal cancer. Incomplete resection of the tumor will lead to recurrence. Palliative treatments such as radiotherapy, chemotherapy, esophageal stents, and gastrostomy are recommended. We insist that the treatment is questionable because this type of LCWEC tumor has no distant metastasis in important organs, so they belong to the locally advanced stage. The selected patients with locally advanced esophageal cancer need to meet the following requirements. The tumor can begin to invade the trachea and aorta, but the tumor did not completely invade the trachea and aorta and could not be resected. Three-dimensional reconstruction of the aorta showed no obvious thickening of aortic branching vessels extending into the esophageal tumor. Fiberbronchoscopy showed no invasion of the posterior tracheal wall and no tumor cells in the tracheal brush. Secondly, patients are with or without supraclavicular lymph node and abdominal lymph node metastasis. Thirdly, the patients have good organ functions and can withstand major surgery. The vast majority of tumor and lymph node enlargement lesions can be resected. Without resection of a tumor, severe complications will arise with the progression of a tumor; the prognosis of tumor patients is poor, with pain and a high risk of mortality. Palliative treatment cannot completely prevent dangerous complications and improve the quality of life for a long time. Palliative chemoradiotherapy might fail, and there is a possibility that radiotherapy causes perforation and infection leading to rapid death. The overall survival of LCWEC patients without any treatment was found to be only 3 months, and none of them survived for more than one year [1]. There might be a residual tumor in the lesion area because of the need to protect the trachea, aorta, and recurrent laryngeal nerve during surgery. Still, adjuvant treatments can destroy the residual tumor cells. We argue that LCWEC is a complicated disease needing urgent management. It is necessary to perform salvage esophagectomy, and the surgery should achieve the overall expected goals: removing the cancerous obstruction, reconstructing the alimentary tract, removing the mass effect and invasion of the tumor, and mitigating the corrosive effect of the digestive fluids. Ultimately, fatal complications (ETF and EAF) can be avoided. Based on the goals above, the surgery purchases valuable time for comprehensive follow-up treatments to improve the long-term survival rate. For esophageal cancer, it is important to understand the real impact of surgery, not only for the palliation of symptoms but for the possibility of long-term local control. Of course, the 5-year survival of patients with regional esophageal cancer is only 25%, or 20% for all stages together [21]. Hence, understanding the long-term impact of local control would require a relatively short time since mortality is high. Future trials should examine this point.

The standard McKeown three-incision esophagectomy begins with intrathoracic esophagectomy, followed by abdominal gastric dissection and cervical anastomosis. Considering LCWEC as a locally advanced tumor, the surgery is exploratory, and we modified the procedure by performing thoracoscopic-assisted small-incision exploration of the right chest at the first stage of the surgery. After the esophageal tumor was resectable, the second stage was abdominal operation after thoracic closure. Zhou and Ninh considered that the mobilization of the 5-cm segment of the lower mediastinal esophagus through the hiatus significantly facilitates the thoracoscopic dissociation of the intrathoracic esophagus [22, 23], and we agree with these modifications. Thoracic esophagus dissociation is a key and difficult step. Therefore, it is important that we first transect the upper and lower ends of the esophagus as traction. During this procedure, the beginning steps are always much easier than the ending ones. We gradually dissociated the most difficult areas of the tumor, such as the involved aorta, trachea, and pericardium.

In this study, there was no case of tracheal membrane and aorta injury. Our experience was that blunt separation of the boundaries between the tracheal membranes, the aorta, and the tumor has to be carried out carefully by the index finger under the sensitive touch of the hands. It is usually not necessary to impose severe consequences on the patients to achieve complete resection.

No case of vocal cord paralysis due to recurrent laryngeal nerve injury was observed in this study. Some lymph nodes are closely related to the recurrent laryngeal nerve, and iatrogenic nerve injury should be avoided. On the one hand, we routinely performed routine preoperative PET-CT examinations. If PET-CT exhibited lymph node metastasis, targeted resection was considered. On the other hand, the boundaries between the lymph nodes and the nerves were gently and bluntly separated by the fingers. We freed the cervical esophagus with the index finger, preventing injury to the vagus nerve and the recurrent laryngeal nerve caused by the equipment’s sharpness and the thermal damage caused by the energy tools.

More than 50% of the patients suffer from functional disorders, mainly due to gastric conduit reconstruction. The most common complications are dysphagia, dumping syndrome, DGE, and reflux [24–26], which seriously affect the postoperative quality of life of patients with esophageal cancer. At present, the value of pyloric drainage is still controversial [27]. Pyloric drainage after esophageal cancer surgery has both advantages and disadvantages [27]. A pooled analysis showed no significant reduction in the incidence of earlier gastric emptying by pyloric drainage [28]. Moreover, non-randomized studies found no correlation between delayed gastric emptying and pyloric drainage [27]. In 2002, in a study conducted by Urschel, pyloric drainage management applied during the esophagectomy decreased the incidence rate of early DGE (*P* = 0.046) [29]. Conclusion inconsistency might be related to the inconsistent definitions of DGE and experimental designs. We argue that restricted pyloric sphincter opening is associated with DGE and gastric content reflux, while excessive pyloric sphincter opening is associated with the dumping syndrome and duodenal reflux. Dysphagia occurs when the anastomotic orifice is too small (narrow), reflux occurs when the anastomotic orifice is too large together, and DGE occurs. The key to preventing functional disorder after esophagectomy is to deal with the size of the pylorus and anastomosis. In this study, there was no case of functional disorder. On the one hand, as a crucial point, we routinely performed partial pyloromyotomy and controlled the pyloric size by limiting the length of the myotomy. On the other hand, we improved the existing anastomotic method, controlled the size of the anastomotic orifice, and increased its circumference and area.

No case of anastomotic leakage was found in this study. Anastomotic leakage might be related to the poor blood supply on both sides of the anastomotic site [30]. In addition, there may be factors of infection around the anastomotic site. We argue that the anastomotic site is a contaminated wound from the beginning of anastomosis, and hence, early drainage is crucial. Of importance, both sides of the cervical anastomotic site were placed with a drainage tube, and only three intermittent loose sutures were used to drain the cervical fluid fully.

The resistance of food entering the alimentary tract is related to dysphagia. In order to make the cervical alimentary tract more consistent with the anatomical position and reduce the resistance of food entering the digestive tract, we modified the surgical technique. After transection of the left sternothyroid, the omohyoid, and the left sternohyoid muscles, the gastric tube was anastomosed with the cervical esophagus behind these three muscles instead of in front of them. In order to restore the anatomical positions and function as far as possible, we sutured the transected muscles again.

There was no case of chylothorax in our study. In order to prevent intraoperative injury to the thoracic duct trunk, it is important that the ligamentous connective tissue of the posterior wall of the esophagus could be ligated during the surgery.

Although various reasons have been explained for choosing reconstruction of the digestive tract during esophagectomy, most surgeons follow their preferences and experience. The factors considered for choosing the reconstruction route include the incidence of postoperative complications and death and the length of the reconstruction route. Available studies are limited and inconsistent [22, 31]. Those who chose the posterior mediastinal route argue that the main driving force of gastric tube emptying after esophagectomy is gravity. An important factor for choosing the retrosternal route is to consider the minimal impact of postoperative radiotherapy on gastric tube injury and the primary esophageal tumor bed exposure. We chose the retrosternal route for two reasons. On the one hand, the squeezing effect of the cardiac beat on the gastric tube promotes its emptying. The retrosternal route is considered to be the best, according to the Sabiston Textbook of Surgery (15th Edition) [32]. Based on our experience, the retrosternal route was selected to reconstruct the alimentary tract without DGE. On the other hand, the mediastinal lymph nodes of the patients in this study were metastatic. In addition to the metastatic lymph nodes, the esophageal tumors had suspicious residues, and thus postoperative radiotherapy therapy was required.

Considering the advantages of minimally invasive esophagectomy [33, 34], we applied the concept of minimally invasive surgery to modify the McKeown approach. On the one hand, because LCWEC is characterized by a large tumor and various degrees of peripheral invasion, it is difficult to resect. Furthermore, energy instruments can easily cause thermal damage to the trachea and aorta. Therefore, we chose a hand-assisted thoracoscopic minimally invasive approach to resect the tumors. On the other hand, we chose to perform a hand-assisted laparoscopic operation to reduce the time and trauma of abdominal surgery.

This study has limitations. The sample size was small because (among all the cases operated on at our center) few tumors grow to > 8 cm and still meet the indications for surgery. In this study, it was extremely difficult to calculate the anastomotic site area accurately. Hence, we will perform an animal study to measure the anastomotic site area accurately. Long-term survival is affected by multiple factors, such as patients’ economic conditions, compliance, and diversity of the comprehensive treatment. Thus, our study did not include long-term survival indicators. Even though it is possible to consider that the surgery improved the quality of life of the patients, mainly because patients who could not eat before surgery were able to eat by mouth after, no standardized questionnaire was routinely used during the study period. Our next step will be to expand the investigation by conducting multicenter, prospective, randomized controlled studies.

## Conclusions

This report suggests that the modified Wu’s esophagectomy is a safe and feasible salvage surgical method for resecting LCWEC. Salvage surgery is necessary for LCWEC to avoid fast death due to ETF and EAF. We modified the traditional order of the McKeown procedure. We performed chest exploration at the beginning of the operation and, after that, performed gastric mobilization. Next, we removed the thoracic esophageal tumor (the most difficult part of the surgery), and the cervical anastomosis was performed last. This modification substantially accelerated the process of giant esophageal tumor mobilization. We modified the methods of anastomosis and pyloromyotomy, preventing anastomotic leakage and DGE. We observed no perioperative complications (including anastomotic leakage, anastomotic stenosis, chylothorax, DGE, vocal cord paralysis, reflux, and dumping syndrome) and 30-day and in-hospital mortality. Still, it is only one group of cases from a single center. We will promote our concept to accumulate further experience. We hope to reveal and summarize the specific advantages of this method and apply the mature, more difficult surgical method of resecting LCWEC to other types of esophageal cancers to deal with the complications.

Modified Wu’s esophagectomy is a safe and feasible salvage surgical method for resecting LCWEC. Salvage surgery is necessary for LCWEC to avoid fast death due to ETF and EAF.

## Data Availability

The datasets used and/or analyzed during the current study are available from the corresponding author on reasonable request.
